# Estimation of PHA concentrations from cell density data in *Cupriavidus necator*

**DOI:** 10.1007/s00253-024-13392-z

**Published:** 2025-01-18

**Authors:** Lena Kranert, Rudolph Kok, Anna-Sophie Neumann, Achim Kienle, Stefanie Duvigneau

**Affiliations:** 1 Institute for Automation Engineering, Otto von Guericke University, Magdeburg, Germany; 2 Process Synthesis and Process Dynamics, Max Planck Institute for Dynamics of Complex Technical Systems, Magdeburg, Germany

**Keywords:** Polyhydroxyalkanoate, *Cupriavidus necator*, Estimation, Optical density, Poly-3-hydroxybutyrate, Poly(3-hydroxybutyrate-co-3-hydroxyvalerate)

## Abstract

**Abstract:**

The production of biodegradable and biobased polymers is one way to overcome the present plastic pollution while using cheap and abundant feedstocks. Polyhydroxyalkanoates are a promising class of biopolymers that can be produced by various microorganisms. Within the production process, batch-to-batch variation occurs due to changing feedstock composition when using waste streams, slightly different starting conditions, or biological variance of the microorganisms. Therefore, reliable and online-capable measurement methods of the product concentration are required to monitor and eventually react to those fluctuations. In this work, we present a flexible approach to determine polyhydroxyalkanoate concentrations based on a simple mathematical model. The data-driven model correlates polyhydroxyalkanoate concentrations with optical densities measured at 600 nm, a widespread, fast, and cheap lab measurement. We found that with different cultivation conditions, the correlation needs to be updated for a reasonable PHA determination during the process. We test this approach for the production of poly(3-hydroxybutyrate) and poly(3-hydroxybutyrate-co-3-hydroxyvalerate) in *Cupriavidus necator* using fructose and propionic acid as carbon sources. This flexible approach allows a simple estimation of PHA concentrations and offers the possibility to further enhance biopolymer production when combined with advanced control strategies.

**Key points:**

$\bullet$ *Development of a simple mathematical model to estimate polyhydroxyalkanoate concentrations.*

$\bullet$ *Optical density measurement is used to determine polyhydroxyalkanoate concentration.*

$\bullet$ *The approach is tested for the production of PHB and PHBV with C. necator.*

**Supplementary Information:**

The online version contains supplementary material available at 10.1007/s00253-024-13392-z.

## Introduction

Our planet increasingly suffers from plastic pollution (OECD 2022). One approach to solving this problem is the increased use of biodegradable alternatives. Promising candidates can be found in the class of polyhydroxyalkanoates (PHA). PHAs are bio-based, non-toxic, and biodegradable with properties similar to conventional petroleum-based plastics and, for these reasons, eco-friendly alternatives to those polymers (Raza et al. 2018). All those favorable properties open a broad range of possible applications, for example, in packaging and coating (Sängerlaub et al. 2019), medical applications (Raza et al. 2018), or food industries (Rydz et al. 2018). PHAs can be synthesized by more than 300 different microorganisms (Grigore et al. 2019). The polymer is synthesized by most of these microorganisms when carbon is in excess and the lack of a nutrient such as nitrogen or phosphorus limits cell growth (Anderson and Dawes 1990). The most studied representative accumulating PHA as an intracellular product also used in this work is *Cupriavidus necator* (*C. necator*).

An important issue during the production of PHA is monitoring the product formation. Monitoring product formation opens the possibility of reacting to changes in substrate concentrations or process behavior. A mathematical model of the product formation also allows optimization of the process or the use of advanced control methods such as model predictive control (Morabito et al. 2019).

The direct online measurement of PHA is not possible, as the bacteria store the produced PHA in so-called granules or carbonosomes in the cytoplasm, making PHA an intracellular product. Therefore, usually, the polymer needs to be separated from the cell to precisely determine its concentration. When using gas chromatography (Furrer et al. 2007; Lee and Choi 1995) or liquid chromatography (Satoh et al. 2016), a pretreatment step with an acid or alkaline methanolysis (Braunegg et al. 1978) or alkaline hydrolysis (Duvigneau et al. 2021) is needed. Those methods are therefore time-consuming and not suitable to monitor PHA formation. Fortunately, suitable methods exist to rapidly determine PHA without the need to extract the polymer. One example is the in-line monitoring of PHA production using photon density wave spectroscopy (Gutschmann et al. 2019). This method, however, requires expensive measurement devices. Another method is the in-situ quantification using fluorescence and side-scatter-based spectroscopy (Kettner et al. 2022). Here, however, cell staining and the need for expensive measurement devices complicate the monitoring.

In the present work, we demonstrate a method to monitor PHA production using optical density (OD) measurements. The OD can be measured with a conventional spectrophotometer and is usually determined during biotechnological experiments to monitor cell growth. We present a workflow that enables the user to determine a simple mathematical model that correlates the PHA concentration with the OD. This workflow allows for a flexible model determination as the model structure is adjustable. We use this method to determine models for two different PHA production processes. These processes cover the production of poly(3-hydroxybutyrate) (PHB) and poly(3-hydroxybutyrate-co-3-hydroxyvalerate) (PHBV), respectively, using *C. necator*. For this, we perform two experiments for each production process. The first experiments, the training experiments, are used to determine the model parameters of different model structures. Suitable model candidates are chosen and then validated using a second test experiment. Those test experiments differ from the first training experiments regarding the process mode and the relevant input variable gas composition, respectively. By doing this, we demonstrate that the presented method can be used flexibly for different PHA production processes. Further, we verify our approach by conducting a cross-validation using the model obtained from the PHB process to estimate the concentrations from the PHBV experiment and vice versa. Finally, we show in a simulation study, how the data-driven model approach can be updated to improve the estimation.

## Methods

### Experimental setup

Synthesis of PHA was conducted by *C. necator* (H16, DSM428, DSMZ GmbH, Braunschweig, Germany). Bacteria were precultured in a 250-mL shake flask filled with 10 vol% LB Medium (Carl Roth, Karlsruhe, Germany). The flask was incubated at 30 $~^{\circ }$C and 150 rpm until an OD of 3 at 600 nm was reached. An appropriate volume of the preculture was used to inoculate the second preculture with an initial target OD of 0.4. The second preculture was grown in a 1-L shake flask containing 10 vol% culture medium until an OD of 3 was reached. This second preculture was used to inoculate a total of 1 L culture medium in a DASGIP parallel bioreactor system (Eppendorf AG, Hamburg, Germany) with an initial target OD of approximately 0.4.

For this work, different reactor experiments were performed. PHB was produced in a batch and a continuous setup. Cells were grown in M81 medium that was modified based on Franz et al. (2011) with an additional 5 g/L to have an initial concentration of 25 g/L fructose (Carl Roth, Karlsruhe, Germany). During the batch process, a 2.5 g/L ammonium chloride shot was given at 32.65 h. For the continuous experiment, a batch starting phase with the same initial conditions as in the previously described batch process was carried out to encourage the growth of residual biomass. After that, the feed rates were set to constant values of 50 mL/h between 12 and 37.5 h and 70 mL/h after 37.5 h. During these continuous phases, the inflow of medium and outflow of reactor contents were kept constant. The medium composition of the feed was identical to the growth medium. During both PHB processes, pH was kept constant at 6.8 by adding 2 M NaOH and 2 M H_2_SO_4_. The dissolved oxygen was kept at 70% and the temperature was 30 $~^{\circ }$C. Stirrer speeds varying from 400 to 1100 rpm and gas flow rates between 6 and 16 sL/h were used to control the dissolved oxygen.

For the PHBV processes, the cells were cultivated in a fed-batch setup. They were grown in M81 medium supplemented with 20 g/L fructose, 1.5 g/L ammonium chloride, and 0.5 g/L propionic acid (Sigma-Aldrich, St. Louis, USA). The cells were fed with 20 g/L propionic acid with a feed rate of 8 mL/h. The temperature was kept constant at 30 $~^{\circ }$C. The initial pH was 6.8, due to the constant feeding with propionic acid the pH changed during the experiment accordingly (Fig. S1). The stirrer speed was 400 rpm, and the air gassing rate was 6 sL/h. Consequently, the DO was uncontrolled during the PHBV processes (Fig. S2). During the PHBV test experiment, carbon dioxide and nitrogen were additionally introduced into the system. The gas composition for both PHBV processes can be seen in Fig. S3.

### Analytical procedures

OD was determined using a spectrophotometer (VWR International, Radnor, USA) at 600 nm (Azubuike et al. 2020). Samples were measured as triplicates and diluted with 0.9% sodium chloride solution to stay in the linear range of 0.1 to 0.3. Additionally, 0.9% sodium chloride solution was used as a blank.

For the determination of cell dry weight (CDW), 1 mL culture broth was centrifuged in pre-weighed reaction tubes for 10 min at 13,000 rpm and 4 $~^{\circ }$C. Subsequently, the cell pellet was lyophilized overnight and weighted. Measurements were performed in triplicates. Measured CDW concentrations can be seen in Fig. S4.

PHB and PHBV concentrations were measured with high-performance liquid chromatography as described in Duvigneau et al. (2021). Measurements were performed in triplicates.

An enzymatic test kit (R-Biopharm AG, Darmstadt, Germany) was used to determine ammonium concentrations.

Fructose and propionic acid concentrations in the supernatant were determined with a Shimadzu high-performance liquid chromatography (HPLC). For fructose, 10 $\upmu$L filtered sample was loaded on a Shim-pack SCR-101C (Shimadzu, Japan), eluted isocratically with 0.6 mL/min H_2_O at $80\,^\circ$C, and detected with a RID at $40\,^\circ$C. For propionic acid, 10 $\upmu$L filtered sample was loaded on a reversed-phase column (Intertsil ODS-3, GL Sciences, Japan), eluted isocratically with 1 mL/min 0.1 M NH_4_H_2_PO_4_ (Carl Roth, Germany) at pH 2.6 and $40\,^\circ$C, and detected with a diode array detector (DAD) at 210 nm.

### Parameter fitting

Model parameters were obtained by minimizing the residual sum of squares (RSS) between the model output and the experimental measurement. The RSS is calculated as follows:

1 $$\begin{aligned} RSS = \sum \limits _{i=1}^{N} (y_i - f(x_i))^2. \end{aligned}$$

The experimentally determined PHA concentrations are indicated by *y*. The values *f*(*x*) are the PHA concentrations that were obtained using the respective model with the experimental OD values *x* and the determined parameter set. The built-in Matlab function +fminsearch+ was used to minimize the RSS given in Eq. 1 to determine the parameter set.

### Model selection

The model’s goodness of fit was compared using the corrected Akaike information criterion (AICc) (Sugiura 1978) and the R^2^. The AICc has the following form:

2 $$\begin{aligned} AICc = N \log \left( \frac{RSS}{N}\right) + \frac{2MN}{N-M-1} \end{aligned}$$

where *N* is the number of data points, *M* is the number of unknown parameters, and *RSS* is the residual sum of squares obtained from Eq. 1.

R^2^ is determined as follows:

3 $$\begin{aligned} R^2 = 1 - \frac{\sum \limits _{i=1}^{N} (y_i - f(x_i))^2}{\sum \limits _{i=1}^{N} (y_i - \bar{y})^2} \end{aligned}$$

where $\bar{y}$ is the mean of the experimental data.

### Cross-validation

We used the mean absolute error (MAE) to compare the goodness of the PHA estimations for the cross-validation section:

4 $$\begin{aligned} MAE = \frac{\sum \limits _{i=1}^{N} \left| y_i - f(x_i)\right| }{N}. \end{aligned}$$

## Results

In this work, we present a workflow to create a mathematical model with the purpose to monitor the PHA concentration based on OD measurements at 600 nm. In the following section, we first describe the workflow and outline the necessary steps. The application of the workflow is then demonstrated and verified within a cross-validation and simulation study using two processes: the production of PHB and the production of PHBV using *C. necator*. In the last two sections, the model structures are verified within cross-validation, and the possibility of a model update is tested in an additional simulation study.

**Table 1:** Candidate model structures for fitting the experimental data

Number	Model structure	Number of parameters
1	$y = ax^b$	2
2	$y = a+b \text {log}(x)$	2
3	$y = a(x/(b+x))$	2
4	$y = a(1-e^{-bx})$	2
5	$y = a-bc^x$	3
6	$y = (a+bx)/(1+cx)$	3
7	$y = a(1-e^{-bx})^c$	3
8	$y = a(1-[1+(x/c)^d)]^{-b})$	4
9	$y = a[1-e^{-(b(x-c))^d}]$	4
10	$y = bx+a$	2
11	$y = cx^2+bx+a$	3
12	$y = dx^3+cx^2+bx+a$	4
13	$y = ex^4+dx^3+cx^2+bx+a$	5

The dependent variable *y* is the PHA concentration, *x* is the OD at 600 nm, and *a*, *b*, *c*, *d*,  and *e* are the fitted parameters. Models 1 to 9 were taken from Flather (1996), and models 10 to 13 are polynomials of various degrees

### Workflow

In the following, the necessary steps to obtain a mathematical model that correlates the OD with the PHA concentration are described. In the first step of our workflow, a suitable experiment must be carried out to obtain a data set for parameter identification of the model. A suitable data set for the development of such a model (training data set) should contain the PHA concentration and OD measurement for different process time points. Further, a narrow interval between the sampling times leads to a model with a higher goodness of fit. Finally, the experimental conditions of the training data set should be similar to those of the final production process, which aims to be observed.

In the second step, a set of model candidates must be chosen. Table 1 illustrates a selection of possible model structures for our example cases. Numbers 1 to 9 were taken from Flather (1996), and numbers 10 to 13 are polynomials of various degrees. However, the model sets are adapted to the use cases of this contribution and should be reformulated or extended when transferring the workflow to other processes. More complex models such as artificial neural networks are also conceivable. However, artificial neural networks require a large amount of data and are computationally expensive. In addition, the selection of a suitable network architecture is often based on trial and error. Given these complexities, a simpler model should be considered first, especially if data or computational resources are limited.

In the third step, the parameters of each model candidate have to be determined by minimizing the RSS as given in Eq. 1. Finally, the resulting best model candidates are compared to select the best overall model. The RSS alone is not a good measure for this model selection, as the models differ in their number of parameters. A lower RSS, therefore, does not necessarily represent a better fit as overfitting might occur. For this reason, we decided to use different additional statistical metrics to compare the goodness of fit of all model candidates. We additionally used the corrected AICc (Sugiura 1978) and R^2^ as described in Eqs. 2 and 3, respectively. A lower AICc value indicates a better description of the data compared to a model with a higher value. An R^2^ value closer to one indicates a better fit.

**Fig. 1:**
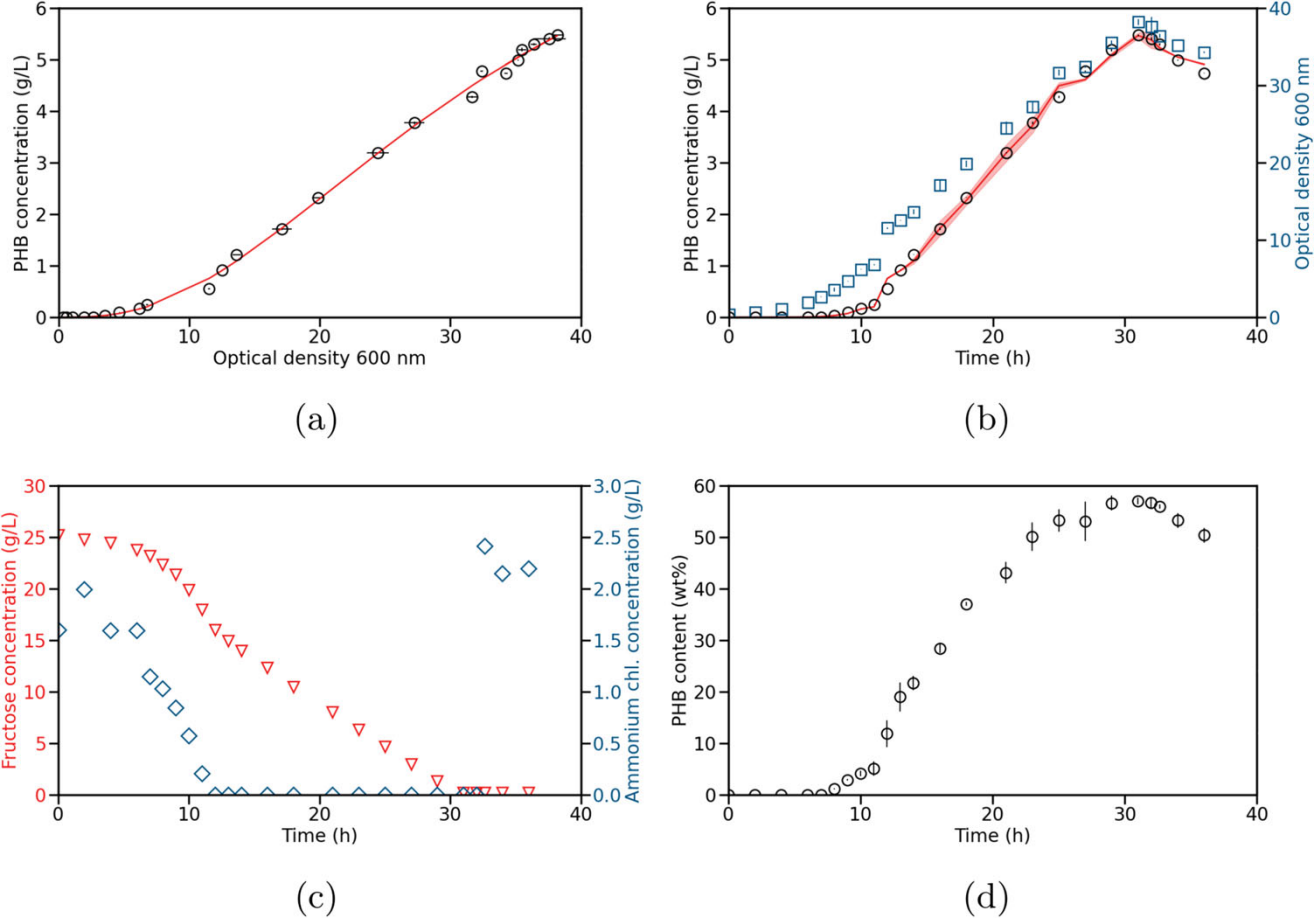
Training batch experiment for PHB production with *C. necator*. Model 7 was used to calculate the simulated values. **a** Measured (circles) and simulated (solid line) PHB concentration over OD measured at 600 nm. **b** Measured (black circles) and simulated (solid line with standard deviation) PHB concentration and measured OD (blue squares) over time. The standard deviation of the simulated PHB concentration was calculated using error propagation. **c** Measured fructose (red triangles) and ammonium chloride (blue diamonds) concentrations over time. A 2.5 g/L ammonium chloride shot was given at 32.65 h. **d** PHB contents (mass fraction of PHA in the cell dry weight) with their respective standard deviation over time

**Table 2:** Statistical metrics calculated for the training data of the PHB and the PHBV production processes

Model	PHB production			PHBV production		
number	RSS	AICc	*R*^2^	RSS	AICc	*R*^2^
1	0.735	$-$31.757	0.994	0.133	$-$49.582	0.988
2	34.907	8.476	0.696	2.776	$-$17.912	0.753
3	4.830	$-$12.139	0.958	0.480	$-$36.206	0.957
4	4.861	$-$12.072	0.958	0.483	$-$36.143	0.957
5	108.557	22.931	0.056	11.221	$-$0.724	0.003
6	0.992	$-$26.006	0.991	0.120	$-$48.001	0.989
7	0.189	$-$43.308	0.998	0.134	$-$46.843	0.988
8	49.129	17.572	0.573	0.181	$-$40.840	0.984
9	67.522	20.887	0.413	6.014	$-$4.319	0.466
10	2.066	$-$20.989	0.982	0.245	$-$43.219	0.978
11	2.779	$-$15.270	0.976	0.390	$-$35.743	0.965
12	0.216	$-$38.986	0.998	0.120	$-$45.130	0.989
13	0.199	$-$36.629	0.998	0.084	$-$45.573	0.993

In the final step, the suitability of the selected model for predicting PHA concentrations based on OD measurements is validated using a second data set (test data set). The general requirements for this test data set are the same as for the training data set. However, the experimental setup for the test experiment needs to differ compared to the training experiment to investigate the robustness of the model to different experimental conditions.

### PHB production

The first process covers the production of PHB using *C. necator*. As a training experiment, a batch cultivation was performed. The process conditions are described in the section “Experimental setup.” During the training experiment, samples were taken every 1 to 3 h. The results of the training experiment are shown in Fig. 1. PHB production could be observed after 10 h of cultivation after the depletion of ammonium chloride. Further, it can be seen that the addition of ammonium chloride at 32.65 h after depletion of fructose leads to the consumption of PHB while the CDW (Fig. S4a) remained the same, therefore increasing the residual biomass. Consequently, the amount of PHB in the total biomass decreased after the addition of the nitrogen source (Fig. 1d).

Using the experimental data for OD and PHB concentration, the parameters of the models in Table 1 were identified by following the workflow from the “Workflow” section. For this, the RSS was minimized. Additionally, the AICc and R^2^ values were calculated for each model. These values can be found in columns two to four in Table 2. Based on these metrics, model 7 captures the trend of the training data best with AICc = $-$43.308 and R^2^ = 0.998. Using the determined parameters, the resulting model has the following form:

5 $$\begin{aligned} c_{PHB} = 8.2031\cdot (1-e^{-0.0556\cdot \text {OD}})^{3.1885}. \end{aligned}$$

The estimated PHB concentrations over OD and over time are illustrated in Fig. 1 a and b, respectively.

To test the limits of the presented approach, a test experiment was performed as a continuous process. In PHB production, sometimes a continuous cultivation is preferred to extend the production phase of the polymer (Atlić et. al. 2011). However, performing a continuous cultivation usually takes a lot of time and effort for the preparation. Therefore, our goal was to first develop the equation on a less time-intensive batch experiment and validate it on a continuous cultivation.

**Fig. 2:**
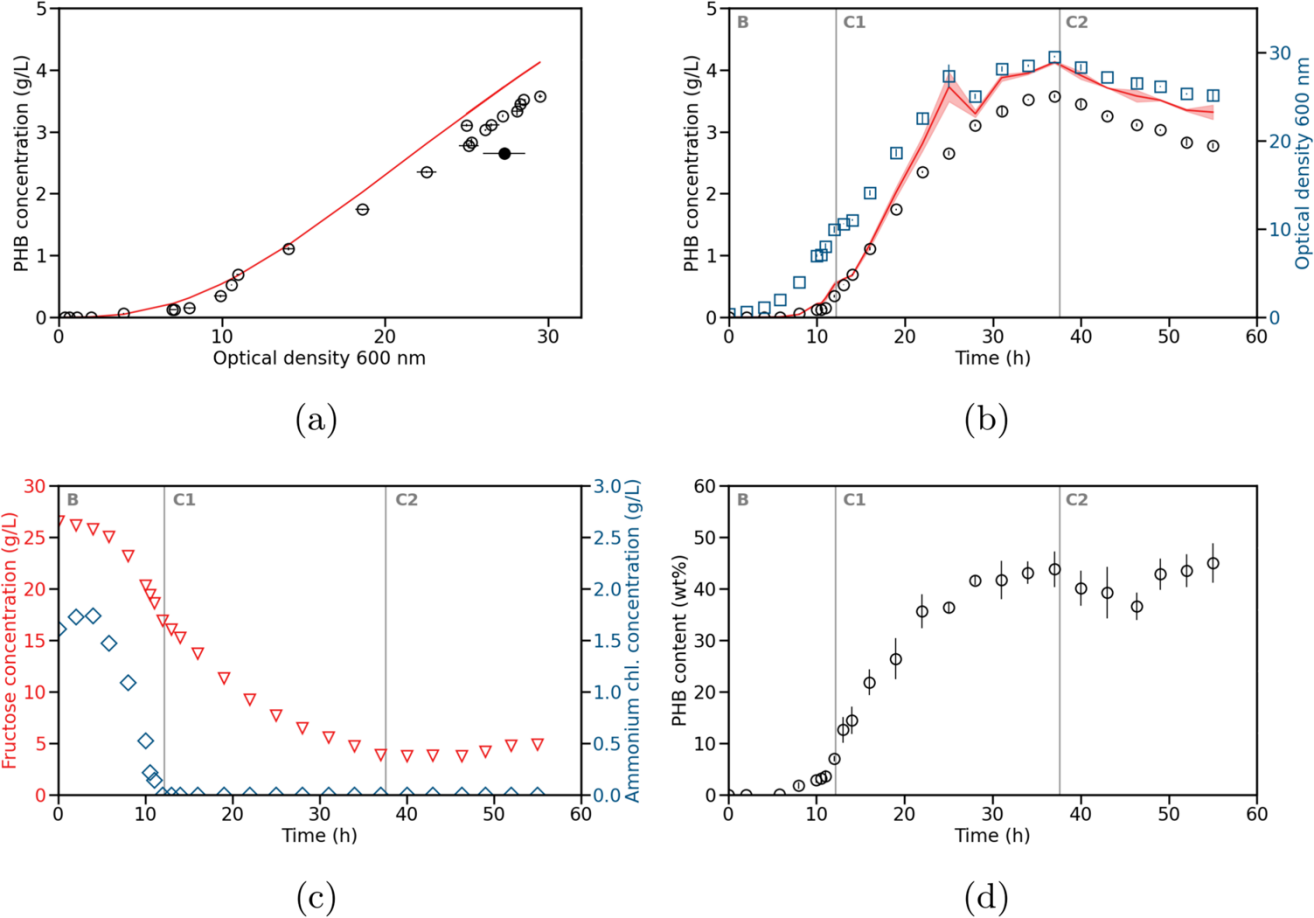
Test experiment for the production of PHB by *C. necator*. The test experiment was conducted in a continuous setup. Model 7 was used to calculate the simulated values. **a** Measured (circles) and simulated (solid line) PHB concentration over OD measured at 600 nm. **b** Measured (black circles) and simulated (solid line with standard deviation) PHB concentration and measured OD (blue squares) over time. The standard deviation of the simulated PHB concentration was calculated using error propagation. The different phases of the process are labeled as B for batch and C1 and C2 for continuous phases one and two with feed rates of 50 mL/h and 70 mL/h, respectively. The start of the continuous phases is illustrated as vertical lines. **c** Measured fructose (red triangles) and ammonium chloride (blue diamonds) concentrations over time. **d** PHB contents (mass fraction of PHA in the cell dry weight) with their respective standard deviation over time

The experimental data of the test experiment are illustrated in Fig. 2. The process started with a batch phase until 12 h. This was followed by a continuous phase with an initial feed rate of 50 mL/h. After 37.5 h, the feed rate was increased to 70 mL/h. The feed composition was identical to the initial medium with 25 g/L fructose and 2.5 g/L ammonium chloride. Ammonium chloride was depleted during the batch phase and was immediately metabolized during the continuous phase. The fructose concentration decreased during the batch and the first continuous phase and remained constant during the second continuous phase.

Using model 7 from Eq. 5, we simulated the PHB concentration of the test data based on the measured OD. As can be seen in Figs. 2b and S5a, the model can reproduce the lower experimental PHB concentrations with high accuracy during the first 20 h of the process. At later process times, the model tends to slightly overestimate the PHB concentrations during the continuous phases. The reason for this could be the difference in process mode between both data sets. The continuous feeding of fructose and ammonium chloride during the test experiment further stimulated the increased production of catalytic active biomass and reduced production of PHB (Fig. S4a). This would then still result in increasing OD values at a lower production rate of PHB, resulting in a deviation between simulated and experimental values. Furthermore, it is already known that cell morphological changes occur with rising cultivation duration and media changes (Tian et. al 2005). Additionally, the number of granules and granule/cell area ratio change with cultivation time (Vadlja et. al 2016). Both factors significantly influence the OD measurement. A change in process mode, especially when conducting longer continuous cultivations, can strongly influence the estimation. However, switching into the second continuous phase (C2) with a feed rate of 70 mL/h, the plant-model mismatch remained constant. Therefore, calculating a $\Delta$PHB based on a measurement during the continuous phase and adding this to the measurement equation might solve this problem. Alternatively, a model update during the continuous process can help counteract this plant-model mismatch. The last results section addresses this solution. Furthermore, at 25 h, there was a significant difference between the model prediction and the measured PHB concentration as indicated by a filled circle in Fig. 2. The likewise increased standard deviation indicates a possible measurement error. This shows that a precise OD measurement is required for a good estimation.

**Fig. 3:**
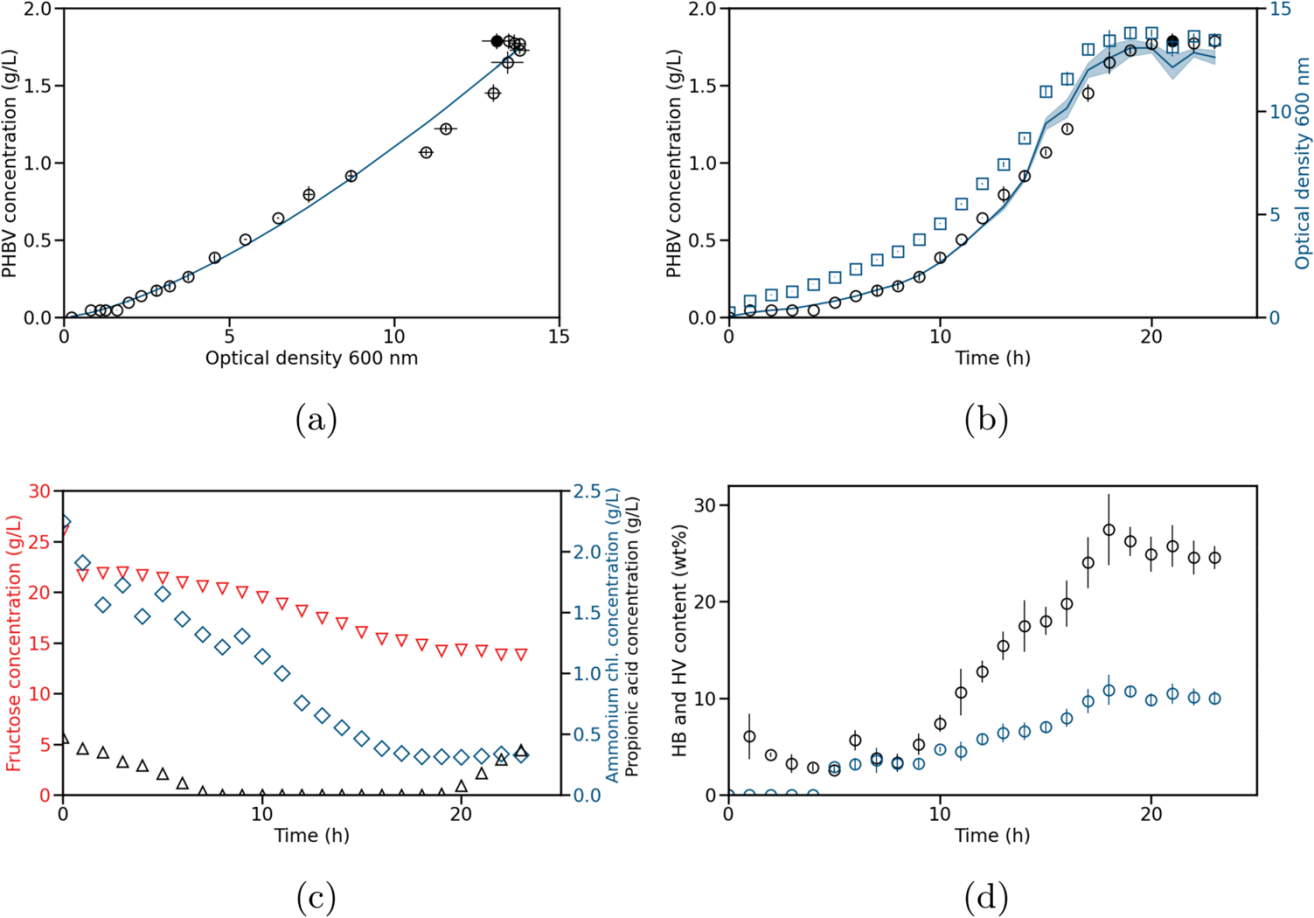
Training fed-batch experiment for PHBV production with *C. necator*. Model 1 was used to calculate the simulated values. **a** Measured (circles) and simulated (solid line) PHBV concentration over OD measured at 600 nm. **b** Measured (black circles) and simulated (solid line with standard deviation) PHBV concentration and measured OD (blue squares) over time. The standard deviation of the simulated PHBV concentration was calculated using error propagation. The filled circle indicates a possible measurement error of the OD. **c** Measured fructose (red triangles), ammonium chloride (blue diamonds), and propionic acid (black triangles) concentrations over time. A total of 20 g/L propionic acid was added during the whole experiment with a feed rate of 8 mL/h. **d** HB and HV contents (mass fraction of PHA in the cell dry weight) with their respective standard deviation over time

**Fig. 4:**
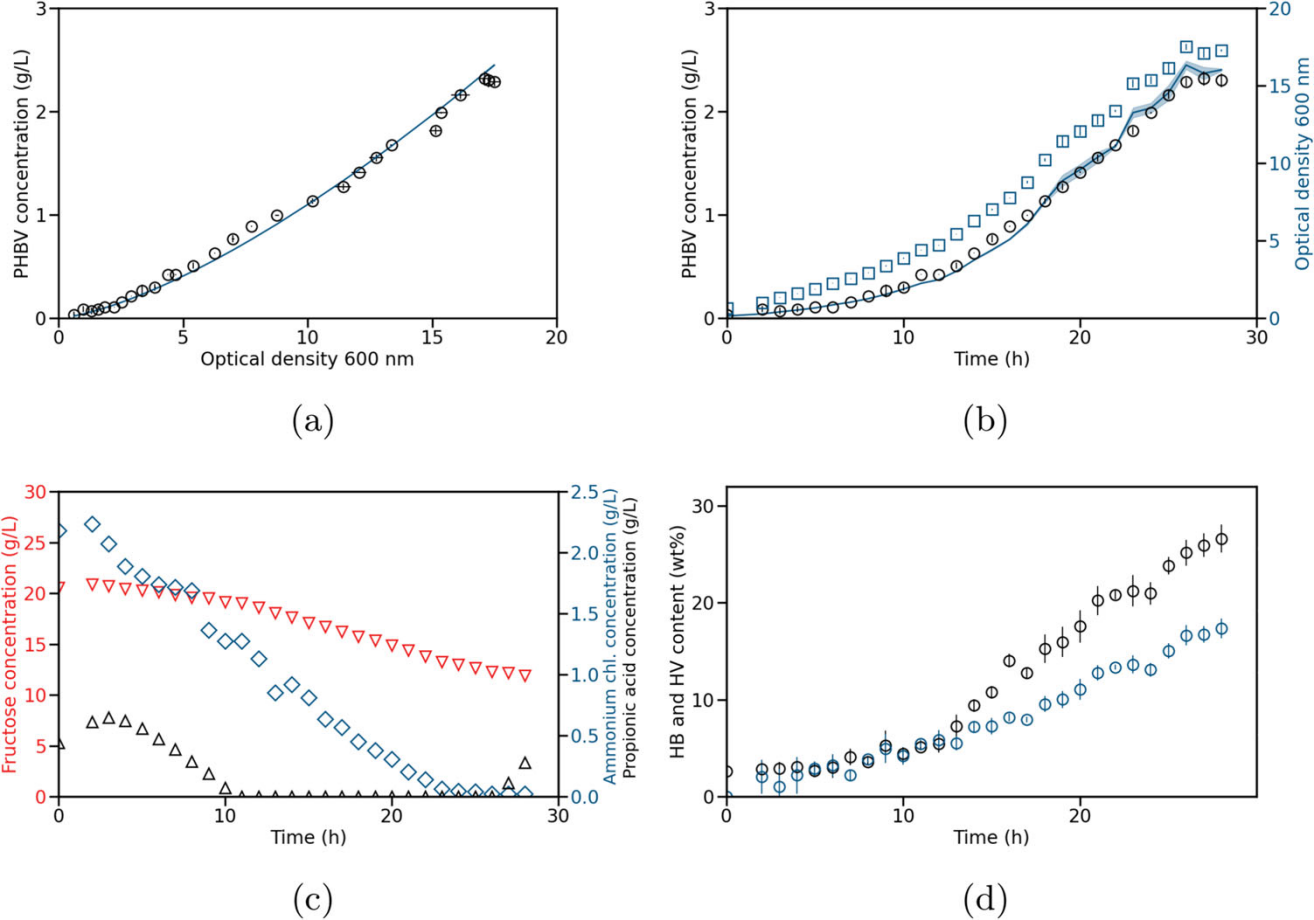
Test experiment for the production of PHBV by *C. necator*. The test experiment was conducted as a fed-batch experiment. Model 1 was used to calculate the simulated values. **a** Measured (circles) and simulated (solid line) PHBV concentration over OD measured at 600 nm. **b** Measured (circles) and simulated (solid line with standard deviation) PHB concentration and measured OD (blue squares) over time. The standard deviation of the simulated PHBV concentration was calculated using error propagation. **c** Measured fructose (red triangles), ammonium chloride (blue diamonds), and propionic acid (black triangles) concentrations over time. A total of 20 g/L propionic acid was added during the whole experiment with a feed rate of 8 mL/h. **d** HB and HV contents (mass fraction of PHA in the cell dry weight) with their respective standard deviation over time

### PHBV production

As the second process, we considered the production of PHBV using *C. necator*. As a training experiment, fed-batch cultivation was performed with propionic acid in the feed. The detailed process conditions are described in the “Experimental setup” section. During the training experiment, samples were taken every hour. The experimental data of the training experiment are shown in Fig. 3. In comparison to the PHB processes, the PHBV accumulation was triggered due to oxygen limitation as can be seen in Fig. S2. Propionic acid with a concentration of 20 g/L was fed continuously with 8 mL/h. The propionic acid concentration in the medium increased and the cells stopped producing PHBV after about 19 h. As well as in the PHB example, the model parameters were identified by following the presented workflow. The RSS, AICc, and R^2^ values were calculated and are illustrated in columns five to seven in Table 2. Based on the AICc, the resulting best model is model 1 with AICc = $-$49.582. When using R^2^ to select a model, model 13 with R^2^ = 0.993 is the best candidate. Due to the similar statistical metrics of both models, we ultimately decided to continue with model 1 due to its simpler structure. Using the determined parameters, model 1 has the following form:

6 $$\begin{aligned} c_{PHBV} = 0.0404 \cdot \text {OD}^{1.4341}. \end{aligned}$$

The estimated PHBV concentrations over OD are illustrated in Fig. 3a. The time courses for PHBV (model prediction and experimental data) and OD are shown in Fig. 3b.

In the following, we want to test our model applying it to a second experiment. In comparison to the training experiment, the inlet gas composition was varied during the test experiment with respect to the amount of carbon dioxide and nitrogen to test the robustness of the approach. The changes in dissolved oxygen and the gas composition are illustrated in Figs. S2b and S3b, respectively.

The experimental results of the test experiment are illustrated in Fig. 4. Similarly to the training experiment, the cells stopped producing PHBV and the concentration of propionic acid increased after about 27 h. When comparing the monomer contents in the total polymer from the training and test data sets (Fig. S6a and b), an increase in the HV content can be seen.

Following our approach and using the before parameterized model 1, we simulated the PHBV concentration of the test data based on the measured OD. As seen in Figs. 4b and S5b, the estimations are in good agreement with the experimental data.

**Fig. 5:**
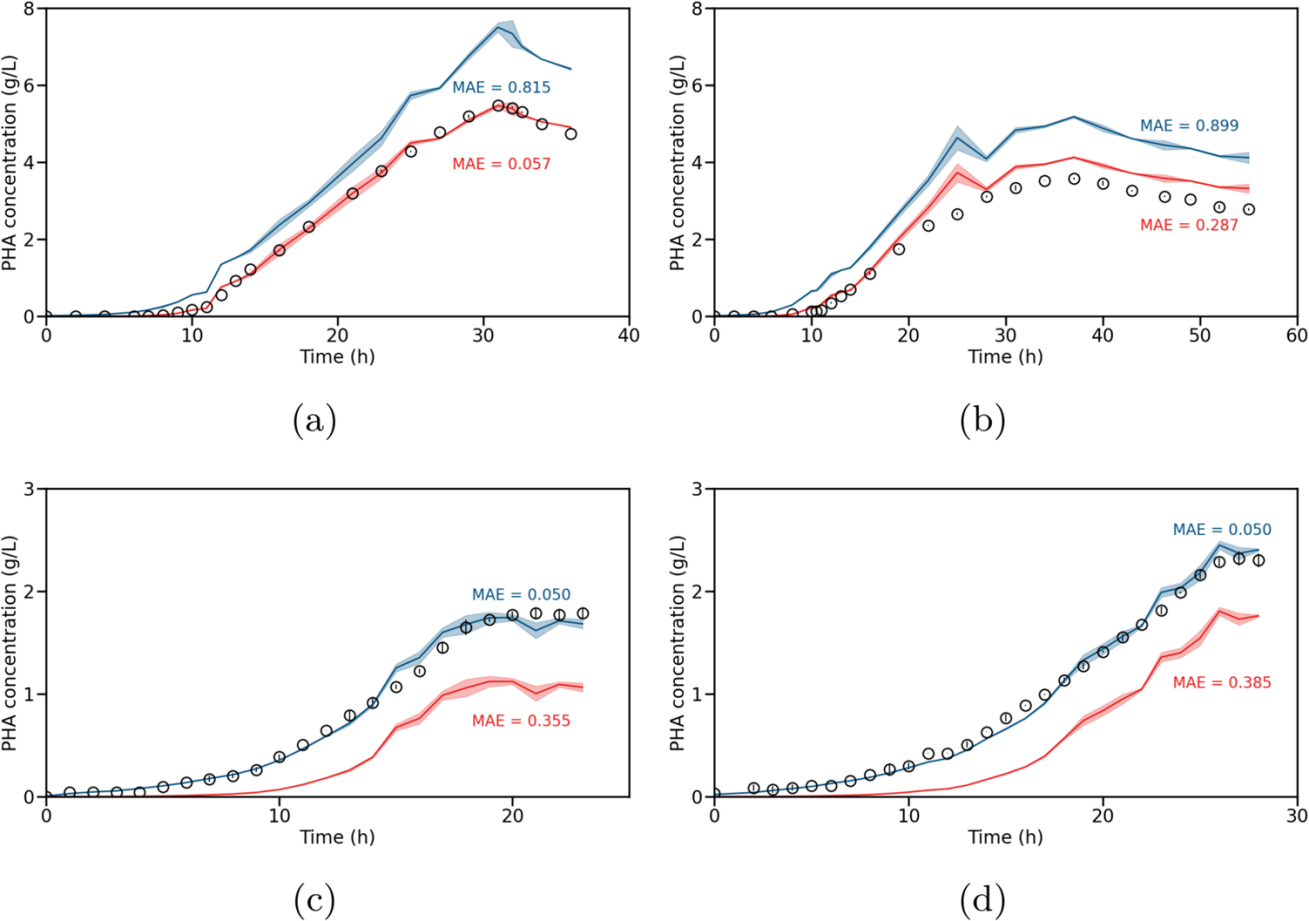
Cross-validation study using the PHB model (red lines) and the PHBV models (blue lines). The measured PHA concentrations are shown as black circles. The respective MAE are plotted in red and blue. The illustrated experiments are **a** the PHB training, **b** the PHB test, **c** the PHBV training, and **d** the PHBV test experiment

### Cross-validation

In the following section, we compare the performance of the PHB (Eq. 5) and the PHBV model (Eq. 6) by estimating the PHA concentrations for all four processes. The results are illustrated in Fig. 5. We used the MAE to compare the goodness of each PHA estimation.

For all processes, it can be seen that the respective model (PHB model, red line; PHBV model, blue line) provides a better estimation. Additionally, it can be seen that the estimations using the PHBV model are higher than the estimations using the PHB model. One possible explanation of this behavior can be found in the application of different process modes when producing PHB and PHBV, which could change the cell in size and granularity and, consequently, the correlation between OD and PHA concentration. Detailed investigations supporting this hypothesis can be found in Vadlja et. al (2016), where an analysis using transmission electron microscopic images for a continuous PHB cultivation with five stages was performed.

However, as already seen in Fig. 2b, the PHB concentrations of the PHB test experiment using the PHB model (red line) are slightly over estimated (Fig. 5a), with an MAE that is five times higher than in the PHB training experiment (Fig. 5b). We suspect that the cause of this overestimation is the change in process mode. Therefore, in the next section, we will investigate whether a model update could improve the model estimation.

### Simulation study: model update

In this section, we investigate whether the PHB estimation of the continuous PHB test experiment can be improved by updating the underlying model equation. For that, we assume that during the experiment, a certain number of past PHB and OD samples can be measured. These measurements are then used to update the model equation. We consider two time points where we update the model parameters: first, after the shift to the first continuous phase after 12 h and, second, after the shift to the second continuous phase after 40 h. For the PHB and OD, all past measurements plus all data points from the training data set are considered. This leads to two extended training data sets with 33 and 44 data points, respectively. In order to focus more on the newly measured data, we have weighted the data from the test experiment by a factor of five. The model updates are delayed to the sampling at 14 h and 43 h, respectively, since the HPLC measurement for the determination of the PHB concentration based on Duvigneau et al. (2021) takes approximately 2 h. For a real-time estimation, we would consider the possibility that only the model parameters are adjusted, which ensures a fast model update. Within our simulation study, we also allowed the model structures to change. However, based on the statistical metrics, model 7 continued to be the best model candidate.

**Fig. 6:**
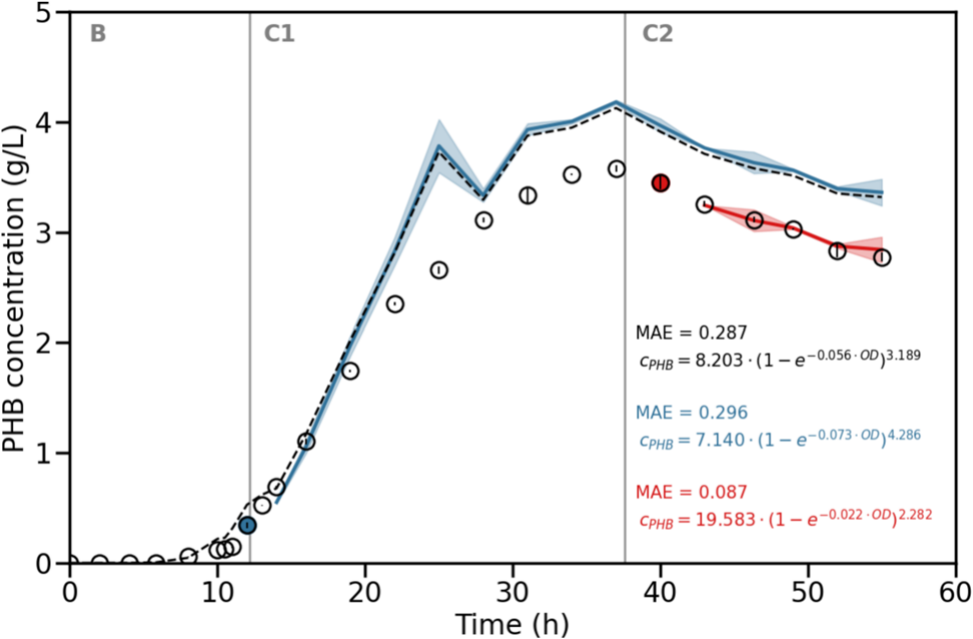
Simulation study to investigate a possible model update during the process. The measured PHB concentrations of the PHB test experiment are shown as black circles. The different phases of the process are labeled as B for batch and C1 and C2 for continuous phases one and two with feed rates of 50 mL/h and 70 mL/h, respectively. The start of the continuous phases is illustrated as vertical lines. The original PHB estimation based on Eq. 5 is shown as a black dashed line. The two time points up to which new measured data were taken into account are shown as blue and red filled circles. The PHB estimations based on the updated model equations are shown as blue and red lines, respectively. The MAE value of each estimation and the corresponding model equation are stated in the respective color

The results of this simulation study are illustrated in Fig. 6. Here, it can be seen that the PHB estimated using the first parameter update (blue line) was similar to the original estimation. This is due to the additional measurements coming from the batch starting phase of this continuous process. However, the PHB estimated using the second parameter update (red line) showed a better prediction compared to the original estimation, because the measurements from the first continuous phase were considered. This confirms the assumption from the “PHB production” section that, due to the same plant-model mismatch in both continuous phases, the data from the first continuous phase can be used to update the parameters for the second continuous phase.

## Discussion

The ability to monitor the PHA concentration is useful for the polymer production process. Such monitoring makes it possible to react to changing substrate concentrations or the production behavior of the microorganisms. Furthermore, monitoring can be used to control and optimize the production process in real time.

In the current work, we presented a simple and flexible strategy to create a mathematical model to monitor PHA formation from the OD at 600 nm at-line. The quality and range of application of the strategy are demonstrated in two microbial PHA production processes. Here, we created tailored measurement equations and validated them.

In the first case shown in the result section (PHB production), we demonstrated that a suitable model equation can be found independent of the fermentation mode since we had changed the mode from batch to continuous fermentation. With the possibility of a model update during the continuous PHA production process, one can improve the estimation with limited measurement effort. In the second case (PHBV production), we changed the gassing composition during the process to validate the model in the context of other possible manipulated process variables. Further, we investigated the possibility of a model update with additional measurement information for the PHB process. All in all, the results show the ability of the approach to determine PHA concentrations even if the fermentation mode or process inputs are changed from training to test experiments. Model updates further improve the goodness of an initial model when changing fermentation modes. Model updates could in future work also be used to include cell lysis and an increased conversion of PHBV into biomass as this was not the focus of this work.

Due to the small amount of time required to measure the OD, the presented method is advantageous compared to conventional quantitative PHA measurements such as gas chromatography (Furrer et al. 2007; Lee and Choi 1995) and liquid chromatography analysis (Satoh et al. 2016; Duvigneau et al. 2021). As only a spectrophotometer is required, this method is also cheaper and easier to establish than other PHA monitoring techniques such as photon density wave spectroscopy (Gutschmann et al. 2019) or fluorescence and side-scatter-based spectroscopy (Kettner et al. 2022).

In comparison to other aforementioned completely independent measurement approaches, it is necessary to determine the best model for predicting PHA considering the equipment (e.g., spectrophotometer, bioreactor system) available in the laboratory.

As already mentioned, the presented method can determine total PHA concentrations. Based on the purpose of the PHA quantification, a separation into different monomers, such as hydroxyvalerate, is desirable. For this purpose, gas chromatography or liquid chromatography methods should be used.

Future work will focus on the investigation of sophisticated model structures like artificial neural networks and on the combination of the presented models with other methods to improve PHA production. One example would be the use of state estimation methods and a process model to reconstruct unmeasured state variables from available measurement information as shown in Carius et al. (2018). In this paper, a preliminary measurement equation based on OD with rather limited applicability was combined with different state estimators to estimate the concentrations of PHA, biomass, fructose, and ammonium chloride. The presented method could, therefore, contribute to improving the upscaling of the process and thus to performing PHA production processes in an economically efficient way.

Furthermore, the presented OD-based method could be transferred to other fermentation systems producing internal products, such as glycogen or cyanophycin. The homopolysaccharide glycogen is formed, similar to PHA, during the excess of carbon under growth-limiting conditions, e.g., because of the lack of a nitrogen source (Preiss 2014). Glycogen is usually measured using fluorometric or colorimetric assays or using a colorimetric estimation with iodine shown by Krisman (1962). Cyanophycin can be formed and used by microorganisms as a storage compound for nitrogen, carbon, and energy and is also formed under limiting conditions, e.g., phosphate limitation (Frommeyer et al. 2016). This molecule is currently quantified using modified versions of the Sakaguchi reaction assay developed by Messineo (1966). These methods are time-consuming and therefore not suitable to monitor the production of glycogen and cyanophycin. Therefore, the presented method could be used to simplify the monitoring of intracellular products. Additionally, a future objective is to transfer the presented approach to PHA production processes under more complex cultivation conditions, such as substrates from waste streams. Furthermore, the presented approach could be combined with a correlation of CDW over OD to estimate the PHA content during PHA production processes.

## Supplementary Information

Below is the link to the electronic supplementary material.

Supplementary file 1 (pdf 692 KB)

## Data Availability

The authors confirm that all data generated or analyzed during this study are included in this article and its supplementary materials.
