# Supplementary material for: Estimation of PHA concentrations from cell density data in Cupriavidus necator
Source: Appl Microbiol Biotechnol. 2025 Jan 18;109:11. doi: 10.1007/s00253-024-13392-z (PMC11742799; doi:10.1007/s00253-024-13392-z)
Supplement: Supplementary file 1 [file 253_2024_13392_MOESM1_ESM.pdf]

Supplementary Material

Applied Microbiology and Biotechnology

Estimation of PHA concentrations from cell density data in *Cupriavidus necator*

Lena Kranert[1*], Rudolph Kok[1], Anna-Sophie Neumann[1], Achim Kienle[1,2], Stefanie Duvigneau[2]

[1] Institute for Automation Engineering, Otto von Guericke University, Magdeburg, Germany.
[2] Process Synthesis and Process Dynamics, Max Planck Institute for Dynamics of Complex Technical Systems, Magdeburg, Germany.
[*]Corresponding author email: lena.kranert@ovgu.de

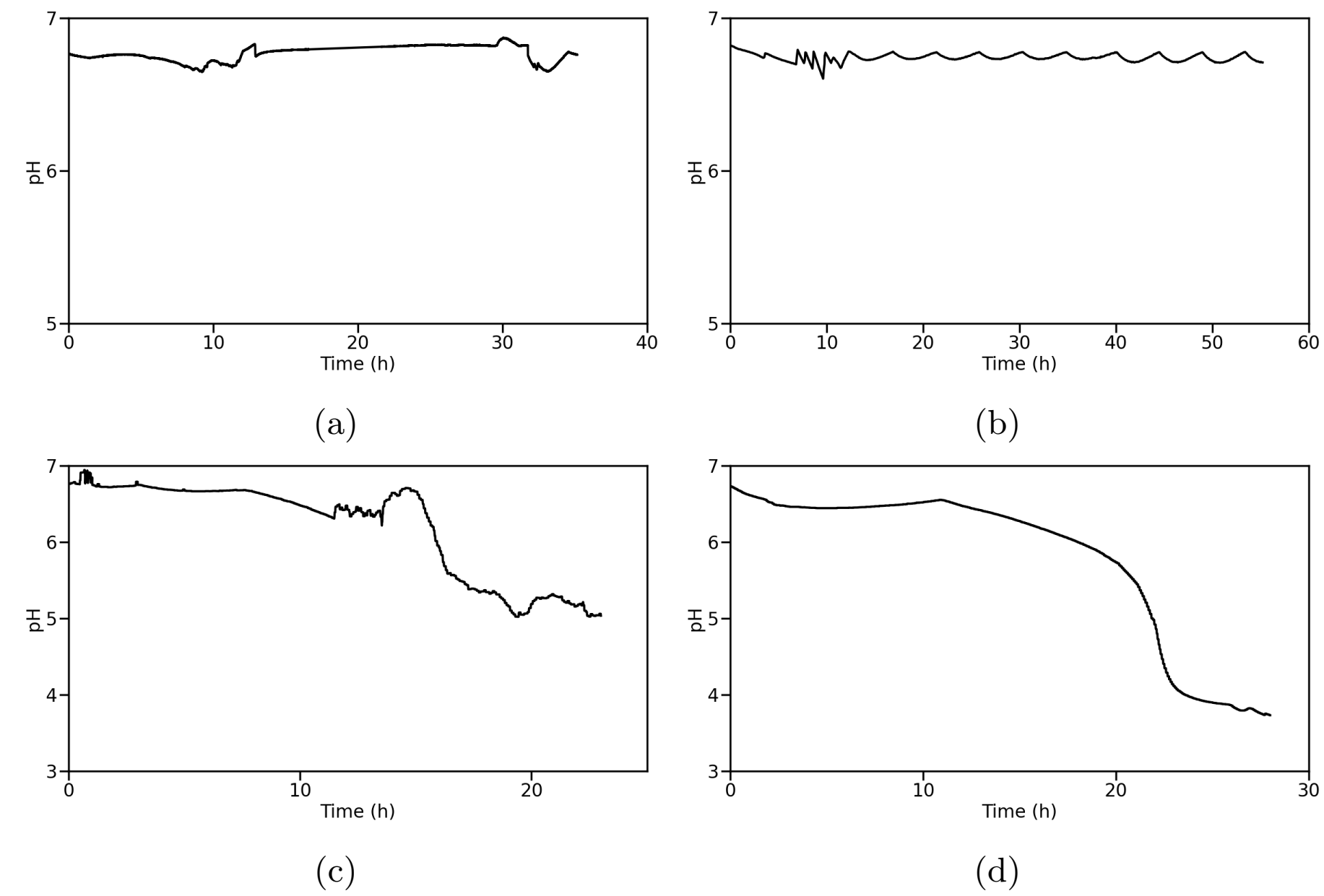

Fig. S1 pH over time. (a) PHB training experiment, (b) PHB test experiment, (c) PHBV training experiment, (d) PHBV test experiment

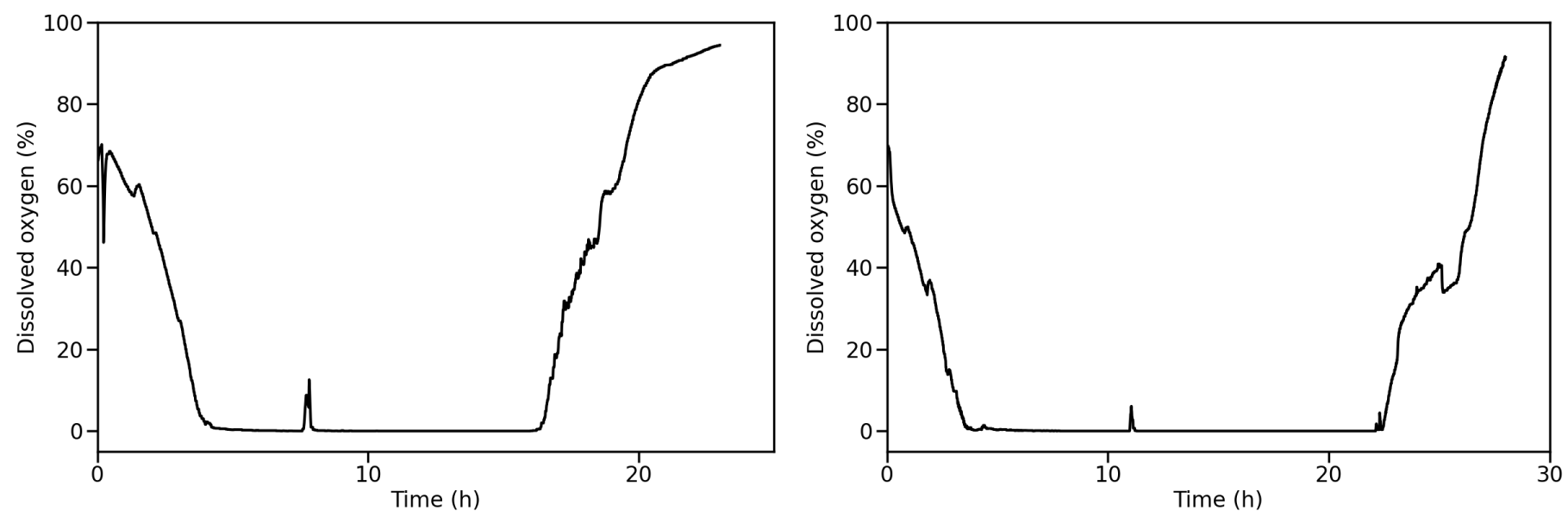

(a) (b)

Fig. S2 Dissolved oxygen over time for the (a) PHBV training and the (b) PHBV test experiment

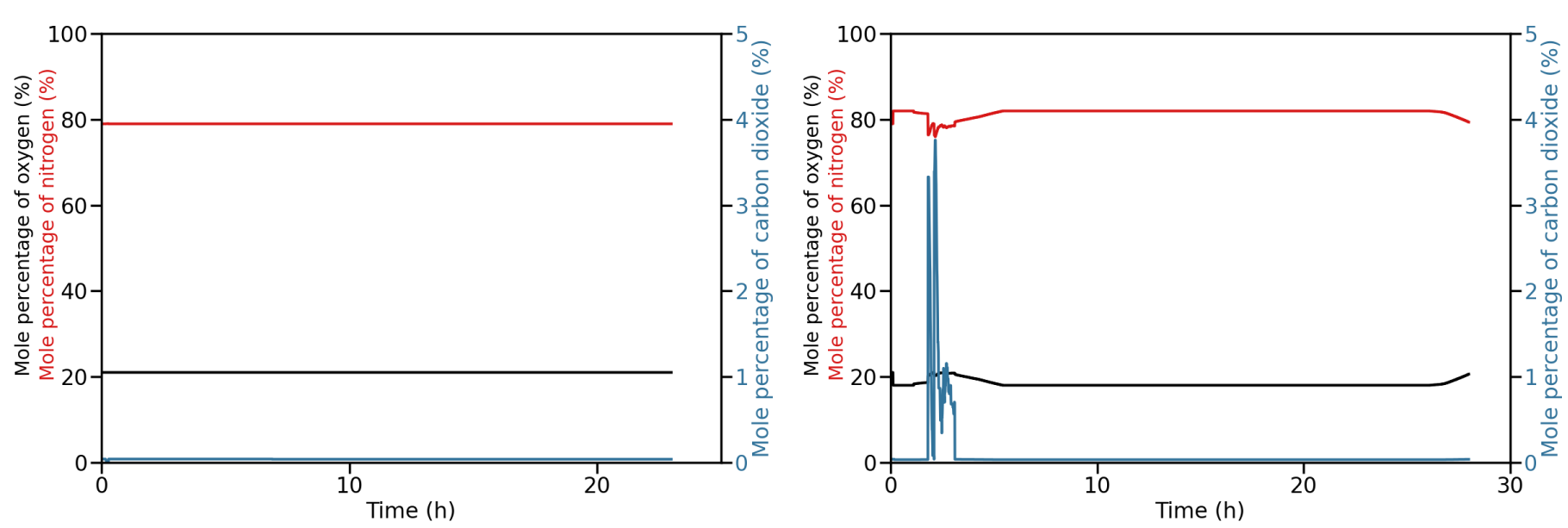

(a) (b)

Fig. S3 Gas composition of inlet gas over time for the (a) PHBV training and the (b) PHBV test experiment. Mole percentage of oxygen (black line), nitrogen (red line), and carbon dioxide (blue line)

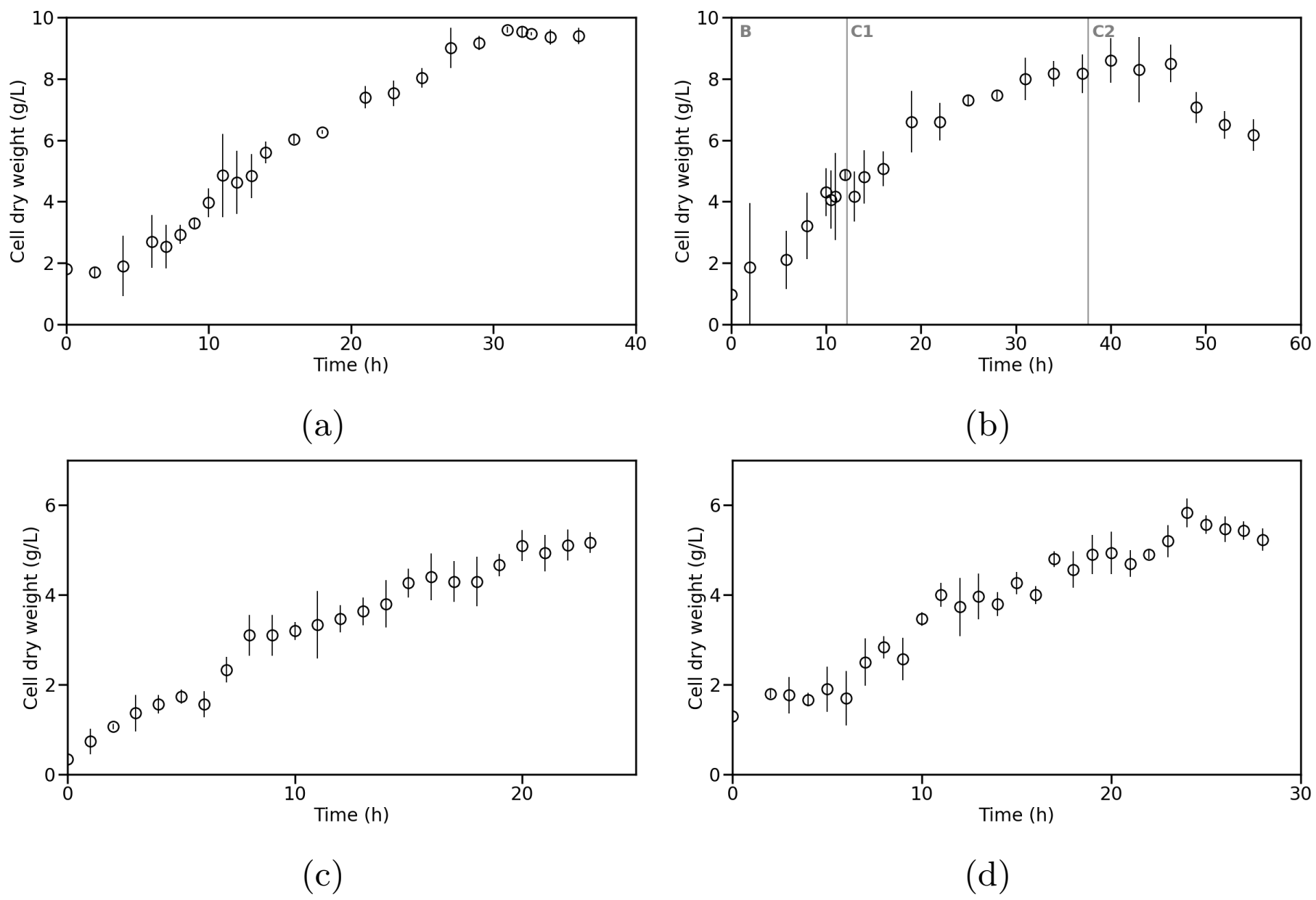

Fig. S4 Cell dry weight over time. (a) PHB training experiment, (b) PHB test experiment, (c) PHBV training experiment, (d) PHBV test experiment

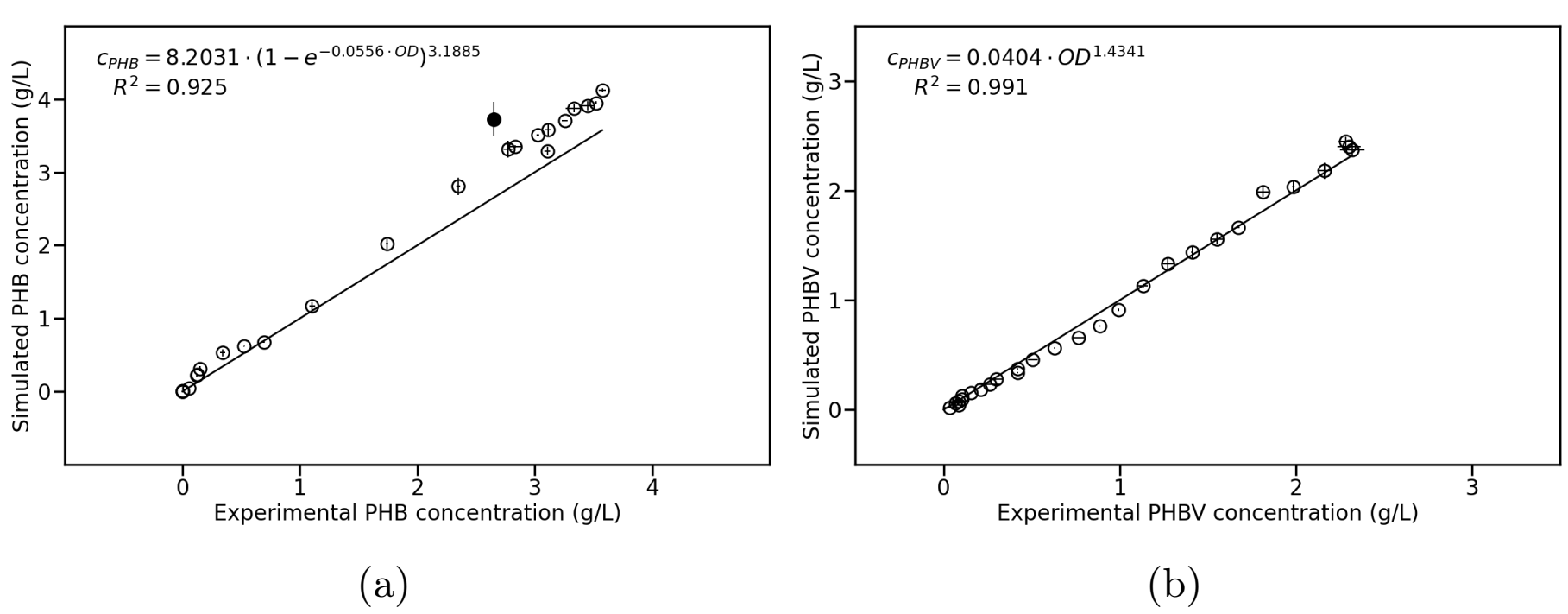

Fig. S5 Parity plots showing the distribution of experimental vs. simulated PHA concentrations. Additionally, the corresponding model equation and R^2 value are shown. (a) PHB test experiment, (b) PHBV test experiment

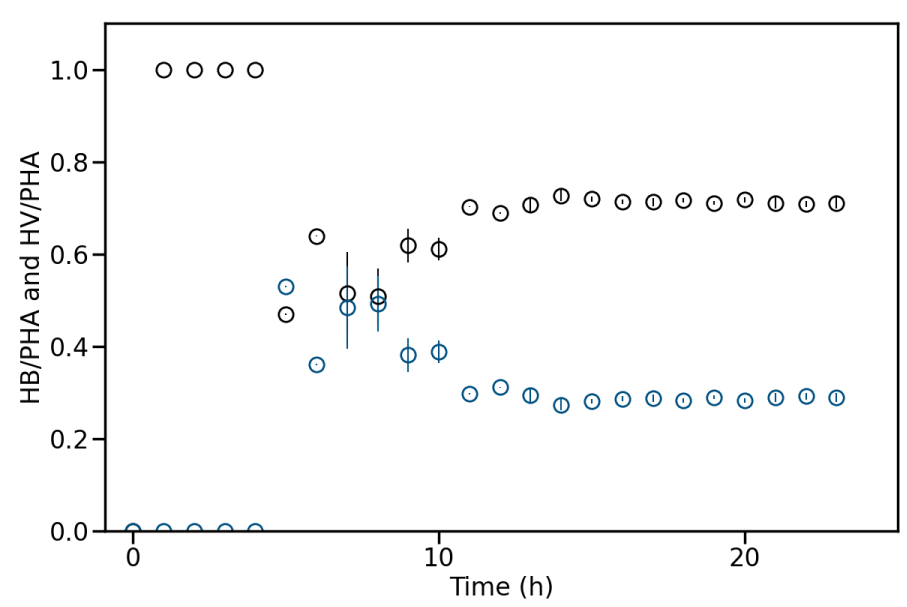

(a)

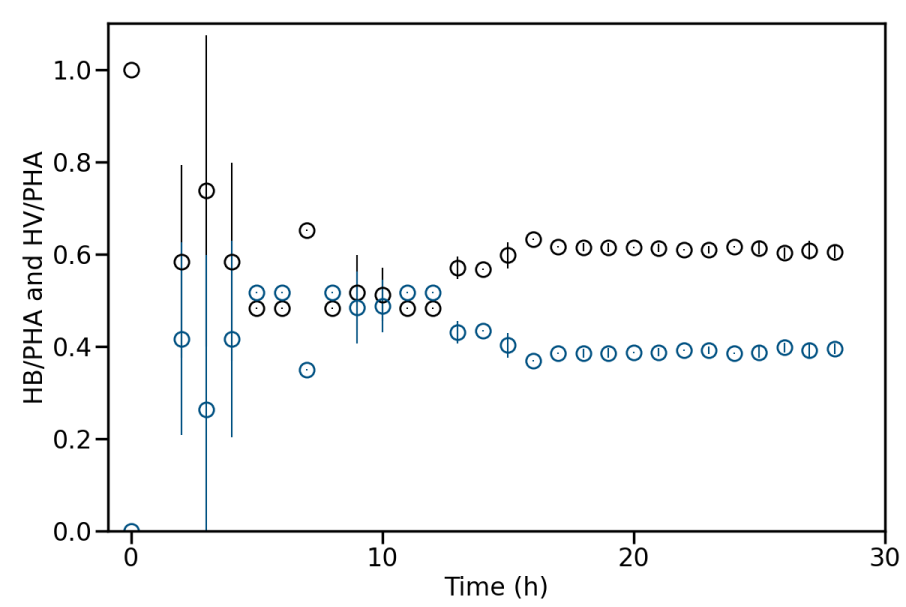

(b)

Fig. S6 HB (black circles) and HV content (blue circles) in the accumulated PHA, respectively, for the PHBV (a) training and (b) test experiment
